# Analysis of applying a patient safety taxonomy to patient and clinician-reported incident reports during the COVID-19 pandemic: a mixed methods study

**DOI:** 10.1186/s12874-023-02057-6

**Published:** 2023-10-14

**Authors:** Thomas Purchase, Alison Cooper, Delyth Price, Emma Dorgeat, Huw Williams, Paul Bowie, Jean-Pascal Fournier, Peter Hibbert, Adrian Edwards, Rhiannon Phillips, Natalie Joseph-Williams, Andrew Carson-Stevens

**Affiliations:** 1 Division of Population Medicine, School of Medicine, Cardiff University, Cardiff, CF14 UK; 2 PRIME Centre Wales, Division of Population Medicine, School of Medicine, Cardiff University, Cardiff, UK; 3 Westway Surgery, Ely, Cardiff, UK; 4 Medical Directorate, NHS Education for Scotland, Glasgow, UK; 5 School of Health, Science and Wellbeing, Staffordshire University, Stafford, UK; 6 Département de Médecine Générale, Faculté de Médecine, Nantes Université, Nantes, France; 7 Australian Institute of Health Innovation, Macquarie University, Sydney, Australia; 8 Allied Health and Human Performance, IIMPACT in Health, University of South Australia, Adelaide, Australia; 9 Cardiff School of Sport and Health Sciences, Cardiff Metropolitan University, Cardiff, UK

**Keywords:** COVID-19, Patient safety, Incident reporting, PISA, Taxonomy, Classification, Systems learning

## Abstract

**Background:**

The COVID-19 pandemic resulted in major disruption to healthcare delivery worldwide causing medical services to adapt their standard practices. Learning how these adaptations result in unintended patient harm is essential to mitigate against future incidents. Incident reporting and learning system data can be used to identify areas to improve patient safety. A classification system is required to make sense of such data to identify learning and priorities for further in-depth investigation. The Patient Safety (PISA) classification system was created for this purpose, but it is not known if classification systems are sufficient to capture novel safety concepts arising from crises like the pandemic. We aimed to review the application of the PISA classification system during the COVID-19 pandemic to appraise whether modifications were required to maintain its meaningful use for the pandemic context.

**Methods:**

We conducted a mixed-methods study integrating two phases in an exploratory, sequential design. This included a comparative secondary analysis of patient safety incident reports from two studies conducted during the first wave of the pandemic, where we coded patient-reported incidents from the UK and clinician-reported incidents from France. The findings were presented to a focus group of experts in classification systems and patient safety, and a thematic analysis was conducted on the resultant transcript.

**Results:**

We identified five key themes derived from the data analysis and expert group discussion. These included capitalising on the unique perspective of safety concerns from different groups, that existing frameworks do identify priority areas to investigate further, the objectives of a study shape the data interpretation, the pandemic spotlighted long-standing patient concerns, and the time period in which data are collected offers valuable context to aid explanation. The group consensus was that no COVID-19-specific codes were warranted, and the PISA classification system was fit for purpose.

**Conclusions:**

We have scrutinised the meaningful use of the PISA classification system’s application during a period of systemic healthcare constraint, the COVID-19 pandemic. Despite these constraints, we found the framework can be successfully applied to incident reports to enable deductive analysis, identify areas for further enquiry and thus support organisational learning. No new or amended codes were warranted. Organisations and investigators can use our findings when reviewing their own classification systems.

**Supplementary Information:**

The online version contains supplementary material available at 10.1186/s12874-023-02057-6.

## Background

The COVID-19 pandemic resulted in major disruption to the delivery of healthcare worldwide [1], causing medical services to rapidly adapt their standard practices. Organisations need to learn from the global body of evidence around the impact of COVID-19 to develop strategies that not only focus on short-term recovery, but on the development of sustainable and resilient healthcare systems equipped to withstand similar future events [2]. Learning how such healthcare system adaptations result in unintended consequences and lead to patient safety incidents is essential to mitigate future healthcare-associated harms [3].

Since the early 2000s, healthcare organisations have increasingly used patient incident reporting and learning systems to learn from patient safety incidents and empirically identify opportunities to improve patient safety [4]. The primary function of these reporting systems is to harness the results of data analysis to guide system improvements [5]. Reporting systems form a vital part of routine monitoring of clinical practice [6] and can improve the quality of care delivered by healthcare services through the identification of common safety issues, helping to prioritise funding and resources, and the development of safety interventions [7].

**Fig. 1:**
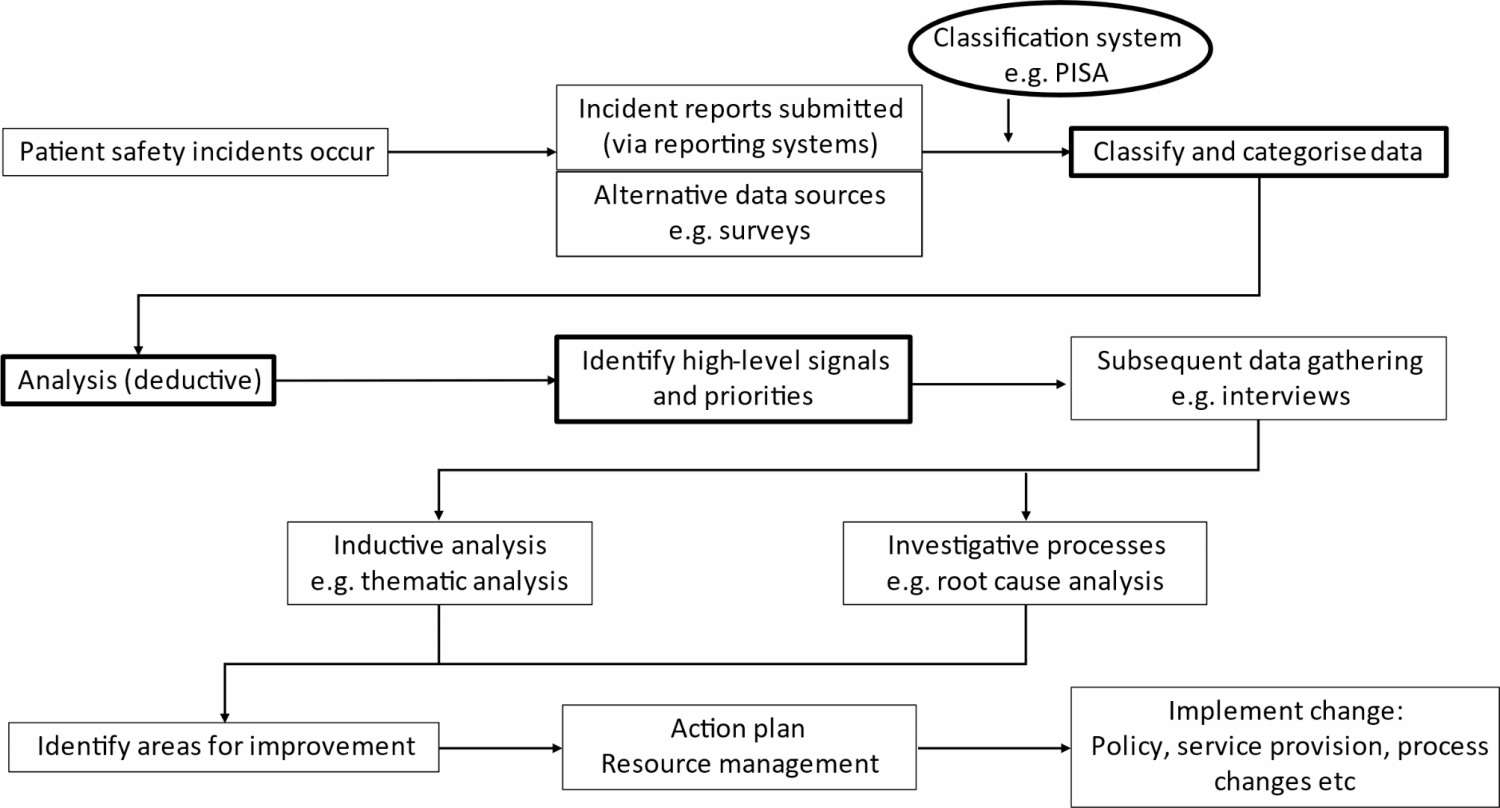
Steps to learning from patient safety incidents. Key steps related to our aims and implementation of the PISA classification system are outlined in bold

Classification systems are important tools for providing structure to meaningful reporting and data analysis [8] and help to make sense of the complex nature of patient safety incidents [9]. A taxonomy is required to standardise these classifications into a hierarchical form to aid categorisation and organisation [10]. Figure 1 illustrates how organisational learning benefits from classification systems.

Previously, inconsistent definitions used in patient safety literature impeded international learning [11]. In 2009, the World Health Organization (WHO) responded to this need to standardise the terms used in classifying patient safety by developing the International Classification for Patient Safety (ICPS) conceptual framework [12]. The ICPS framework allows for greater comparisons to be made between countries and care settings because of a more organised, accepted, and understandable classification inclusive of concepts with standard definitions [13]. In 2014, in response to a lack of granular patient safety classification systems suitable for primary care, our group empirically developed the ‘PatIent Safety (PISA) classification system’ [14] which is ontologically aligned with the ICPS framework. PISA has since been used to analyse more than 75,000 primary care incident reports [14].

The longevity and value of a taxonomy is determined by how well it meets the end-user’s needs [10]. Maintaining this requires the taxonomy to undergo regular review to ensure its coding remains inclusive and comprehensive, yet flexible, allowing for new codes to be added or for existing codes to be amended [15]. The International Classification of Diseases (ICD) provides a common language for healthcare professionals and is a global standard for the systematic recording of morbidity and mortality data [16]. In response to the COVID-19 pandemic, the WHO classification and terminologies unit activated emergency codes for COVID-19 in ICD-10 and ICD-11, regarding its diagnosis, complications, and vaccinations [17]. It is not known whether existing patient safety classifications, like PISA, are sufficient to capture novel safety concepts arising from systemic constraints related to the pandemic in the context of patient incident reporting and learning systems. It is unclear whether they require updating to incorporate COVID-19-specific coding in relation to healthcare-associated harm.

We sought to critically review our experience of using the PISA classification system during the COVID-19 pandemic, with a focus on its ability to classify incidents, enable the initial deductive analysis, and identify areas for further enquiry, thus generating learning from patient- and clinician-reported safety concerns (as shown in Fig. 1). We sought to identify whether modifications to our PISA classification system should be considered to maintain its meaningful use and ensure accessible and timely learning from patients and healthcare professionals.

## Methods

### Study design

We conducted a mixed-methods study integrating two phases in an exploratory, sequential design:

1. A comparative secondary analysis of patient safety incident reports from two studies conducted during the first wave of the COVID-19 pandemic: (i) patient-reported incidents from the United Kingdom (UK); (ii) general practitioner (GP)-reported incidents from France.
2. Presentation of findings to a focus group of health services researchers, clinicians, and leaders.

### Description of reference framework (PISA) and its development

The PatIent SAfety (PISA) Research Group at Cardiff University has led an extensive characterisation of patient safety incidents reported by healthcare professionals from primary and secondary care settings. This includes studies to identify priority areas for patient safety across the health and social care continuum, including unsafe discharge from secondary to primary care settings [18], incidents experienced by children in primary care [19], older adults [20], patients receiving palliative care [21], advanced care planning [22] and adults receiving opiate replacement therapy [23]. The PISA classification system has also previously been used to characterise the nature of patient-reported safety incidents in primary care settings from the UK and Australia, enabling the data to be used for service learning and improvement [24]. The PISA classification system was developed using a constant comparative method [25] on an initial sample of 13,600 reports [26] followed by nine years of iterative development (i.e., addition of new codes, refinement of new and existing definitions). The PISA classification system is used by health services researchers in the UK, Canada, Brazil, France, Spain, and Australia. It incorporates multiple coding frameworks with four independent classes describing the incident (‘what happened?’), the contributing factors (‘why it happened?’), the resultant harm outcome and the level of harm. Each code and its definitions within each class are intended to be ‘mutually exclusive’.

Where an existing code is not available to describe a salient feature within a report narrative, our research group discuss whether a new code is needed, or whether the definition of an existing code should be amended to be more inclusive. This is based on analytical memos created by coders during the review and application of codes. Our interdisciplinary group is comprised of clinicians (doctors, nurses, pharmacists, optometrists, dentists), mixed methods researchers, and Human Factors and patient safety experts.

**Fig. 2:**
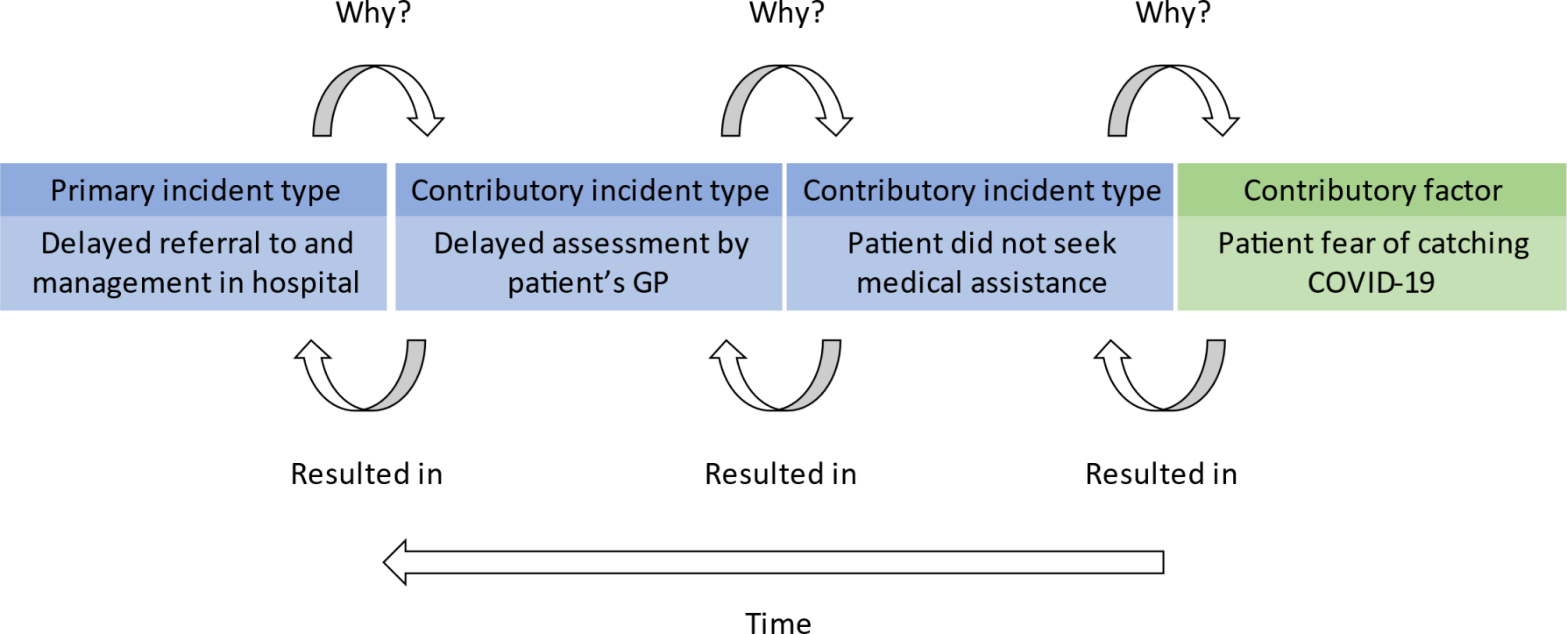
Example of the recursive model of incident analysis

By following the Recursive Model of Incident Analysis (Fig. 2), PISA can capture the chronological sequence of events typically described by reporters as leading to a safety incident. The coding framework is structured into higher-level ‘parent codes’, such as ‘2. Staff factors’ and branches into more nuanced ‘child codes’, such as ‘2.2 Task a piece of work to be done or undertaken’ leading to ‘2.2.1 Failure to follow protocol - failure to adhere to procedures or regulation’. Coders can assign parent or child codes dependent on the explicit content of the incident report.

### Patient- and clinician-reported safety incidents

#### Data sources

The first source of data came from the COVID-19 UK Public Experiences (COPE) study [27]. This prospective, longitudinal study was developed to gain a better understanding of health behaviours, experiences and well-being outcomes throughout the pandemic. The baseline survey was launched on 13th March 2020 and closed on 13th April 2020. The patient cohort was recruited either via social media (using multi-faceted sampling methods) or via two emails sent to participants of Health Wise Wales (HWW), a national population survey and research register of participants who live or receive healthcare in Wales [28]. A three-month follow-up survey was conducted in June/July 2020. This survey included optional, additional modules, one of which was a free-text module for patients to report any ‘healthcare experiences (including patient-reported safety concerns)’. A total of 318 people opted to complete this module.

The second source of data came from an exploratory, mixed-methods study of GP-generated patient safety incident reports relating to COVID-19 in France (henceforth referred to as the PSI RECORd study) [29]. The team created a national patient safety incident reporting platform to collect data on patient demographics and a free-text description of the incident. The contact list of the Collège National des Généralistes Enseignants (CNGE, a French scientific society of general medicine) was invited to participate via email on 28th April 2020 (with one reminder email sent on 28th May 2020). The GPs were asked to report patient safety incidents observed since 17th March 2020. Reported incidents were included up to 29th June 2020. A total of 103 GPs submitted between one and four anonymised reports, generating 132 incident reports for inclusion.

All data from both datasets were fully anonymised, with no personal identifiers remaining within the reports, prior to being added to the PISA platform for coding.

### Data coding and comparative analysis

The anonymised reports from the PSI RECORd study were translated from French into English using Google Translate [30]. A French-speaking member of the team (ED) sense-checked all the translations and generated a list of French-specific abbreviations for reference. Trained coders applied the PISA classification system to code the anonymised free-text information within the COPE (AC) and PSI RECORd (AC, TP) incident reports. Using the principles of the constant comparative method, we undertook an exploratory descriptive analysis of the coded data to identify the most frequent primary incidents and created cross-tabulations to explore the relationships between these incidents, their respective contributing factors, and the harm outcomes. A primary incident was defined as ‘an issue, complication, or lack of something perceived important during a task or process of care delivery, occurring prior to the outcome and is the incident most proximal to the identified patient outcome’ [12]. To ensure validity and reliability of the coding, Cohen’s kappa statistics were calculated for the primary incident type, aiming for a kappa > 0.7, which is consistent with previous similar studies [31]. Drawing on the established methods used to inductively amend the PISA classification system through regular review, the analysis sought to identify potential areas where the classification could be iterated to incorporate new or amended codes or their definitions.

### Expert discussion

Expert review and feedback on the analysis of the COPE and PSI RECORd data were sought through an online focus group. The purpose of the focus group discussion was to identify similarities and differences between codes required to characterise COVID-19 reports and, as a research collaboration experienced in classification development, to recommend whether and how the existing frameworks could be modified to capture relevant learning arising due to unanticipated major systemic constraints like those posed by the COVID-19 pandemic. The participants (Table 1), selected for their expertise in the development and maintenance of classification systems, were invited to attend via email and signed an electronic consent form before taking part.

**Table 1:** List of expert group participants (n = 6) and facilitators (n = 2)

	Position and experience	Interests
Participant 1 (P1)	National programme director for safety and quality improvement	Chartered Human Factors and Ergonomics specialist and has led reviews into safety taxonomy and incident coding frameworks for healthcare settings
Participant 2 (P2) Facilitator	Academic GP	Involved in the development of the PISA classification system and its application within an extensive range of health services research projects
Participant 3 (P3)	Health services researcher and incident analyst/investigator	Has led reviews into safety classification and incident coding frameworks and helped develop the WHO ICPS
Participant 4 (P4)	Senior academic with expertise in patient care improvement	Interest in person-centred and value-based approaches in routine healthcare and patients’ experiences of routine care
Participant 5 (P5)	Academic GP with patient safety research expertise	Involved in the development of the PISA classification system and its application within an extensive range of health services research projects
Participant 6 (P6) Facilitator	GP specialty registrar trained in Human Factors	Has worked on patient safety research projects using incident reporting frameworks to codify data
Participant 7 (P7)	Academic GP with patient safety research expertise	Led the development of the PISA classification system
Participant 8 (P8)	Academic GP with patient safety and quality improvement research expertise	Quality and safety of health care shared decision-making expert

The participants were presented with the findings of the comparative analysis and seven content-rich safety incident examples with different incident types, including the free-text and coding (such as those presented in Table 2). During data analysis, a list of codes was created that were considered to represent new concepts in the context of COVID-19 and suggested new codes which included COVID-19 as a direct contributing factor in relation to the incident. Examples of suggestions included ‘Shielding patient’, ‘Service unavailable due to COVID-19’, ‘Patient fearful of attending healthcare service’ and ‘System change due to crisis management planning’. Guided by the incident examples, codes generated from the secondary data analysis, and semi-structured questions, the expert group discussed whether the PISA classification system warranted iteration to incorporate COVID-19-specific coding (see Appendix 1 for meeting schedule).

The group was facilitated by two of the researchers (AC, TP). The discussion was recorded via Zoom, lasted 1 h 30 min, and was transcribed verbatim in-house (DP).

**Table 2:** Coding unstructured (free-text) data – Patient and clinician examples

Source of incident	Free text	Coding
Patient-reported	*‘I was shocked & outraged to observe that staff with face masks hanging off one ear or under their chin. Totally unprofessional touching their faces with the gloved hands that were worn for whole shift with no use of hand gel or hand washing’*	*Incident type*: Staff professionalism *Contributing factor(s)*: Staff behaviour and infection control protocols *Harm outcome*: No outcome described *Harm severity*: Unclear
GP-reported	*‘The patient called us for vomiting still bloody, despite our repeated advice to call 15 [emergency services for life-threatening conditions] they did not go. The patient died of a digestive hemorrhage 7 h after the first call. The patient’s daughter was afraid to take her to the hospital for fear that she would catch COVID 19 there’*	*Primary incident*: Delayed diagnosis Contributing incident: Environmental hazard *Contributing factor*: Behaviour – patient/family *Harm outcome*: Clinical deterioration *Harm severity*: Death

We then conducted an inductive thematic analysis [32] of the expert group transcript to identify their perspectives on the ability of the PISA classification system to classify incidents from the context of the pandemic, support the initial deductive analysis and identify areas for further enquiry to derive learning. Researchers (DP, TP) ensured thorough familiarisation with the data and generated initial themes based on the purpose and objective of the analysis. These themes were reviewed both individually and during subsequent team meetings. Once the themes had been agreed upon, they were defined and named, and a focussed literature search was undertaken to determine whether these concepts have been described previously in the literature.

## Results

**Fig. 3:**
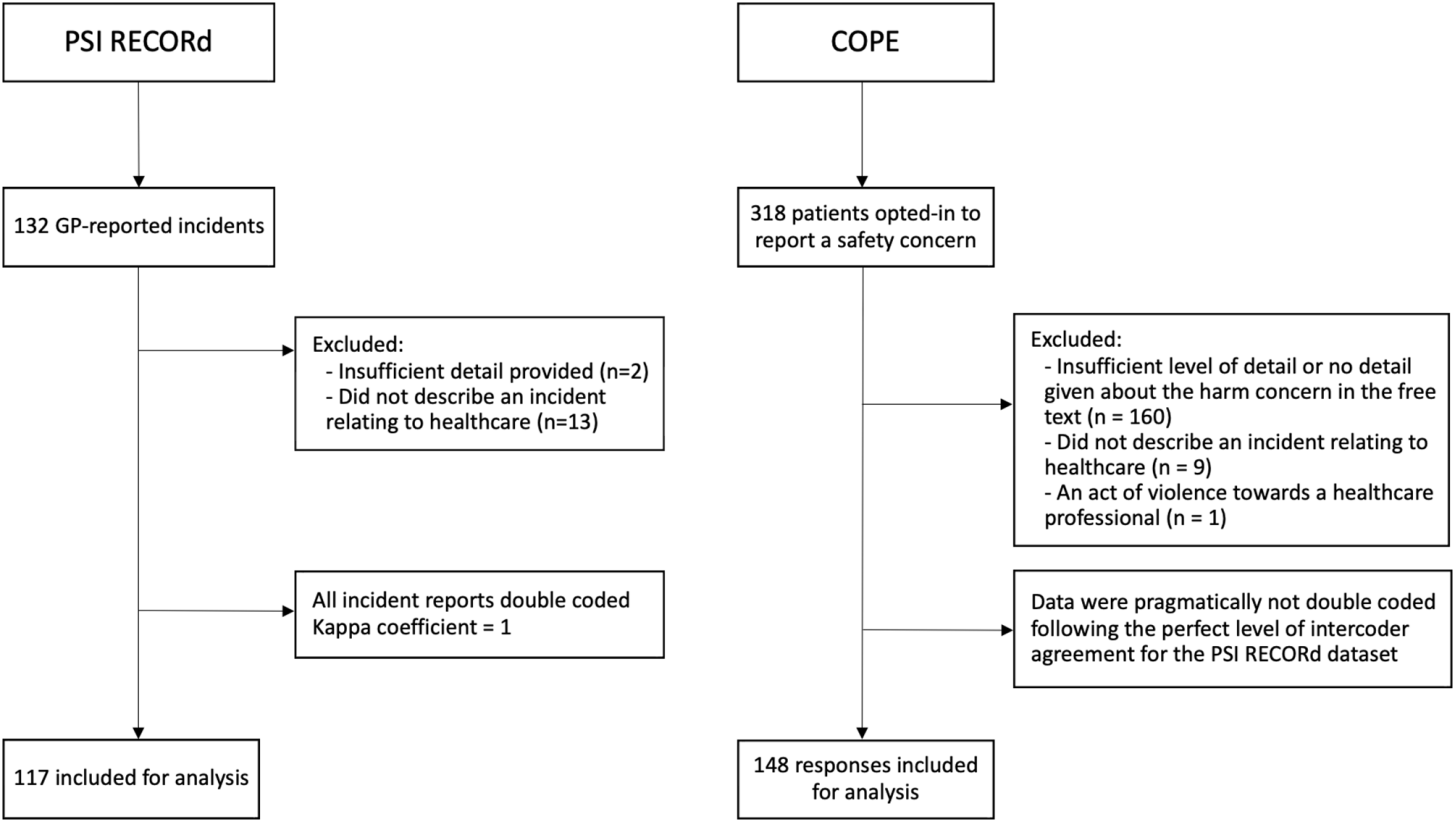
Flowchart of processing clinician- (PSI RECORd) and patient-reported (COPE) incident reports

The breakdown of included and excluded reports from the PSI RECORd and COPE studies are detailed in Fig. 3.

### Thematic analysis of expert group discussion

We identified five themes from thematic analysis of the expert group discussion transcript (Table 3). To help illustrate the context of these themes, they are presented alongside the salient excerpts presented to the expert group from the secondary analysis of the coded data.

**Table 3:** Key themes derived from the expert group

Theme	Description
1. Capitalise on the unique and contrasting lens on patient safety offered by different groups	Patients and clinicians/healthcare professionals may have complementary and differing perspectives on safety
2. Existing frameworks should already enable the identification of high-level signals to inform decisions about where to prioritise resources for further analysis or investigation	Current coding frameworks can capture relevant learning. Be clear on how to generate those signals, e.g., by reviewing the most frequent incident types and severity combinations
3. Consider the objectives and purpose of the inquiry when using the framework, with a process to enable a timely and insightful analysis	Study objectives are key to interpreting the data. The need for granular coding approaches depends on the purpose of the question being asked of the different data sources
4. Be aware of factors that might always have been present but have not been previously captured	Avoid overlooking new learning from less-cited contributing factors that have been brought to the fore in reports in specific contexts like COVID-19
5. Consider the temporal relationship between the period of data collection and substantive events/system constraints	The timeframe in which data have been collected sometimes makes it unique and may offer explanations for new findings. The time periods of major systemic constraints can also be used to contextualise findings rather than generating bespoke labels or new classes (e.g. COVID-19 related) in a classification system

### Clinicians and patients may have complementary and differing perspectives on safety

About half of the patient-reported incidents comprised the two most common incident types: (1) environmental hazards (n = 39/148, 26%), for example, not being able to socially distance, and (2) difficulty accessing healthcare (n = 39/148, 26%), for example cancellation of routine clinics (Table 4). In 21 (14%) reports the primary incident related to staff professionalism and a lack of equipment, such as Personal Protective Equipment (PPE) (example in Table 2).

It was noted by the expert group that these codes have been less frequently used in previous studies, highlighting the learning opportunity now gained when exploring the patients’ perspective. Contrastingly, in the GP-reported incidents, environmental hazards accounted for only one incident and staff professionalism did not feature. This may reflect what patients prioritise as safety concerns, describing events more personal to them or directly related to their immediate surroundings, e.g., PPE and social distancing. The expert group commented on the value gained from these new and varied insights.

*P2: It’s what’s visible to patients as well, so they can see that, but they might not be able to see that their referral has gone missing or they had a badly labelled sample, it’s what they can see, what the people in front of them are doing.*

The GP-reported incidents found that a delay in diagnosis and assessment accounted for 44% (n = 52/117) of the primary incident types. These incident reports specifically related to a delay in the diagnosis in 92% (n = 48/52) of the reports. The next most frequent primary incident type concerned treatment and procedures (n = 21/117, 18%), illustrating the GP concerns were predominantly centred around management of patient conditions.

**Table 4:** Most common Primary Incident Types

Patient-reported incidents	n (%)	GP-reported incidents	n (%)
Access to healthcare	39 (27)	Diagnosis and assessment	52 (44)
Environmental hazard	39 (27)	Treatment and procedure	21 (18)
Staff professionalism and equipment	21 (14)	Investigation errors	17 (15)

### Current coding frameworks can capture relevant learning

Half of the incidents relating to a delayed diagnosis reported by GPs captured ‘patient behaviour’ as a contributory factor (n = 24/48) and most cases (59%) resulted in a delay in patient management. When exploring patient behaviours contributing to the delay in diagnosis, a recurring theme was a fear by patients or their family of presenting to either a GP or hospital setting. This fear stemmed from being exposed to or contracting COVID-19 (example in Table 2).

The underlying contributing factors behind these behaviours are not captured by the current framework. Some GPs postulated that this fear may have been triggered by media coverage in relation to the pandemic, for example. The PISA classification system and its related frameworks potentially does not detect the nuance required, and the expert group agreed that *‘we need to acknowledge the limitations of incident reporting…in terms of the depth you can collect’ (P3)*. However, the PISA classification system identified ‘patient behaviour’ as an area worthy of exploring in more detail. Similarly, when considering the patient-reported examples, the group discussed that the PISA classification system was able to pick out the high-level signals of key broader concepts and concerns. They concluded a meaningful use of a taxonomy should be to enable timely analysis of existing coding that could prompt more detailed follow-on investigation (i.e. interviews with staff and patients, or encouraging more reporting on a specific salient issue) or an inductive analysis of reports with similar characteristics (i.e. a review of all reports assigned the code ‘patient behaviour’ for a specific time period).

*P1: it needs some investigation into why staff are behaving this way, is it because of the inadequate fitting of face masks, they’re too warm, they need to breathe, they feel restricted. These are contributory factors in my head, as to why these types of incidents are taking place. And I don’t know whether, in terms of the reporting we want to try and encourage the reflection on some of these contributory factors. It’s this whole thing about trying to understand, more deeply, the nature of why incidents occur, and designing the classification system to reflect that.*

### Study objectives are key to interpreting the data

About two-thirds (64%, n = 23/46) of patient-reported incidents with a primary incident type of environment hazards had ‘infection control policy’ as a contributing factor, such as the patient example in Table 2. This example only captures the perspective of the patient and the true contributory factors explaining why this incident occurred cannot be fully ascertained from the report. If the aims of this study included identifying the causes for infection control protocols not being followed, or whether an appropriate protocol was in place, then the coding would be inadequate. If the purpose of the study is describing pandemic-related incidents and identifying areas for deeper analysis (as shown in Fig. 1) to derive learning, then the classification is sufficient to accomplish this.

*P2: The depth and the granularity of the coding framework really depends on what you want to do with it. If…you’re just trying to get a handle on what are the main issues here when we’re dealing with a new disease that we don’t know how to manage, then you just want high-level [intelligence] to get an idea about what’s going on.*

### New learning from less-cited contributing factors

Patient factors were also shown to be a contributing factor, but to a lesser extent, in one of the commonest patient-reported incidents concerning environmental hazards (n = 7/39, 18%). A recurring theme for these patient-related contributing factors was fear of presenting to a healthcare setting. The expert group discussed the likelihood that similar concerns around attending healthcare settings for fear of developing a healthcare-acquired infection have always been an important factor but with the context of an infectious pandemic they have been accentuated.

*P2: I would probably suggest that what people end up reporting often reflects some of their existing concerns or worries about healthcare. So, it’s interesting that the patients said a lot of stuff around the doctors not washing their hands or not wearing masks properly, whereas the doctors/GPs are saying ‘well they can’t get access to secondary care in time’, or ‘there wasn’t a service for the patients to come and see us about’. Those are the sorts of things that have been worrying people for a while, before they got an opportunity to report.*

The context of ‘fear’ as a patient behaviour, more specifically fear of contracting COVID-19, posed the question as to whether this warranted an iteration to the existing framework. The expert group considered that generating additional codes to cover multiple eventualities meant that codes may go unused, and may result in a larger and more complex classification system, reducing its usability.

*P3: I think that works better than just continually developing more and more codes, because you can just go on forever.*

### The timeframe in which data are collected sometimes makes that data unique

The commonest contributory factor for an environmental hazard incident type in the patient-reported incidents related to infection control policies. The expert group discussed that the type of infection involved (e.g., MRSA, pneumonia) could impact on the incident and outcome severity. The severity of the incident is only captured by the chosen harm outcome and severity, for example, primary incident type: environmental hazard, contributing factor: infection control protocols, harm outcome: infection, harm severity: death. An ‘infection control policy’ as a contributing factor also does not highlight what the specific concern might be with the policy, or lack thereof. However, an awareness that these data were obtained during the first few months of the COVID-19 pandemic gives a context whereby these concerns may be explainable. For instance, was a new infection control policy being developed or were there policies in place related to crisis management strategies? As the pandemic, our understanding of it, and its impact on healthcare systems was in a state of flux from an organisational perspective, this should be borne in mind when looking at the data.

*P5: It’s really helpful to put it back into the context of where those PRCs [incidents reports] came from, and the setting and timing of when they were reporting. There were lots of new rules coming out about PPE, social distancing…maybe that’s why they were reported, because of the concerns going around at that moment.*

Due to the ever-changing response and re-organisation of healthcare systems during the pandemic, it is pertinent to note the point at which the data were collected since the pandemic began. This is likely to play a role in reports made by both patients and clinicians.

*P4: The timing, this was three months into the pandemic, obviously services had been delayed already to some extent, but maybe at that point they [patients] weren’t really thinking about the impact of that delay on treatment. Or if there was a delay in diagnosis, they probably wouldn’t have even been aware of it at the time.*

Bringing together the above themes, there was a consensus amongst the group that no new codes needed to be added or amended within the frameworks as a direct consequence of COVID-19-related system constraints.

*P5: It’s been helpful to think about where we need to add codes… but the feeling I’m getting back from the group is that these shouldn’t be COVID-specific codes, but [to continue with] more general codes that can then be analysed in more detail from thematic analysis and inductive analysis.*

## Discussion

### Main findings

The meaningful use of the PISA classification system has undergone scrutiny following its application to patient safety incident reports generated during a global pandemic, from healthcare systems where resources were constrained and in which both patients and healthcare professionals faced new challenges and uncertainty [33]. A key finding from our study was that despite these significant system changes, the consensus was the PISA classification system and its frameworks could be meaningfully applied to safety data like incident reports without the need for new COVID-19-specific codes to be added or amended. This suggests that our existing codes can describe healthcare-associated harms which might arise from future system constraints generated by other pandemics or major system stressors, as a basis for further enquiry and analysis. Necessary updates to such frameworks through consistent iterative mechanisms are part of standard implementation.

Our results offer the perspectives of both patients and clinicians at the early stages of the pandemic, which may indicate the types of concerns and the capability of patient safety incident reporting and learning systems that may arise from evolving and future pandemics or other major system stressors. We describe five themes arising from the expert group which organisations and investigators can consider when evaluating and using their own patient safety taxonomies in the context of other major events resulting in substantive system-wide constraints.

### Context of existing literature

Collecting patient safety data from distinct groups enables an opportunity to capitalise on their individual perspectives and gain a broader understanding of how events, such as the COVID-19 pandemic, differentially affects them. Whereas patient or family perspectives may have previously been viewed predominantly as a litigation issue, they are increasingly seen as playing an important role in educating healthcare providers on where and how to improve systems and mitigate against further safety incidents. All patient concerns should therefore be treated equally with healthcare provider concerns, to overcome the blind spots apparent if solely relying on healthcare professional reporting [34]. In keeping with our first theme, previous studies have found both primary and secondary care patients identify and report different types of safety incidents when compared with healthcare professionals [24, 35, 36] and may report incidents that would otherwise go undetected [37]. Engaging patients in patient safety can result in positive outcomes (for patients and organisations) and mitigate the risk of future adverse events [38].

The pandemic is a unifying context for interpretation of both patient- and clinician-reported incidents. The significant disruption and delays to healthcare services, such as routine surgery [39] and cancer care [40], account for patient concerns around access to healthcare and GP-reported delays in treatment and procedures. These system disruptions and reduced service availability heighten patient anxiety regarding disease progression [41], therefore an awareness of any shared concern between patients and clinicians could focus resource management in tackling this area as a priority.

The pandemic resulted in increased levels of anxiety amongst patients [42] with significant numbers avoiding routine and emergency medical care due to concerns surrounding COVID-19 [43, 44]. This concern was captured in the GP-reported incident contributing factors. However, creating a new code for ‘fear of COVID-19’ is likely to be too specific. A trade-off is required between having the right depth to identify important common themes which guide further investigation without requiring too much detail in the coding system. This is consistent with the key concepts that led to the development of ICPS [13]. If the higher-level signals can identify areas to prioritise for further enquiry, then a classification system does not require modification to capture specific incidents related to other system constraints.

Within the scope of our study aims and objectives, the framework did not require modification. However, given the PISA classification system is used across healthcare settings internationally, there may be specific issues that do warrant additional or amended codes. For example, McFadzean et al. [45] explored safety incidents reported from the prison setting in the UK. During this study, new codes within the contributory factors framework were generated, including ‘security priorities’ and ‘lockdown rules (e.g., situations in which prisoners are not allowed out of their prison cell)’, in order to capture learning specific to the context of prison healthcare systems. The new codes were iteratively developed, agreed upon by the research team and will benefit future research investigating prison healthcare.

Substantive events, such as the COVID-19 pandemic, create an opportunity for lesser-known contributing factors to be brought to the forefront of reporting. Such concerns are likely to have affected patients for a significantly longer period without being addressed. For example, codes for incidents relating to a patient’s care environment have previously been used [46] and are therefore not specific to the pandemic. Anxiety surrounding hospital attendance for fear of catching a hospital-acquired infection is also not new, and has been reported with other infections, such as MRSA [47].

### Implications for practice, policy, and future research

To realise the full potential of existing patient safety classification systems and related frameworks and how they can be applied to maximise learning from patient safety incidents during significant system disruption, the following recommendations can be derived from the themes established by the expert group.

Multiple sources of patient safety data are available. When collecting data from only one source there is potential for incidents to go undetected [48]. Our results demonstrate the value added of using different data sources (e.g., patient-completed surveys and incident reporting by clinicians) to generate learning from patient safety incidents. Whilst accepting that there are limitations in terms of the variable depth of data collected through some methods, including patient surveys [49] and incident reporting [50], it is important to ensure that these data continue to be collected and used to maximise the learning opportunities from them. Common themes can then be explored in more detail to elicit a deeper, more nuanced understanding through other methods, such as patient interviews, which can identify additional concerns [51].

The higher-level granularity of data gathered in this study enabled learning in relation to safety incidents during the pandemic. Such signals identified in a similar way during future substantive events can be applied to initiate more in-depth, timely investigative processes (e.g., root cause analysis) which can support the design of improvement projects to reduce the frequency of safety incidents [52]. Exploration of the multi-faceted nature of patient safety incidents, guided by findings from incident reports, should be routinely assisting organisations to understand where and how to implement appropriate change (as illustrated in Fig. 1).

Better reporting systems for receiving and responding to patient concerns are needed and organisations could employ newer methods of data collection, such as the national platform being developed in Wales to gather patient-reported outcome measures (PROMS) [53]. Patients are then offered valuable, additional opportunities to express their experiences in a more structured manner across multiple healthcare settings.

### Strengths and limitations

The international impact of COVID-19 meant analysing two sources of data allowed us to explore a wider scope of safety concerns from both healthcare professionals and patients from two separate countries experiencing the same crisis. This adds to the external validity of our findings and produced a more thorough examination of the framework.

Limitations within the datasets include the quality of data gathered from the patient-reported concerns, as free-text responses were often brief and lacked detail. This may be a result of the question being an optional module amongst a larger questionnaire and without structured guidance on accurately describing a safety concern. The healthcare professional data reported GP concerns only, thereby missing incidents from other community-based professionals. Fournier et al. [29] did attempt to mitigate against this by conducting an ancillary study of pharmacists, nurses, physiotherapists, and midwives, but participation in this study was extremely low with only one incident reported.

We gained valuable insights from an international group of experts in patient safety with a working knowledge of the PISA classification system and its functionality. This provided consensus feedback from end-users on the application and performance of the PISA classification system amidst the pandemic. However, the expert group did not include a Patient and Public Involvement (PPI) representative. Our results miss the additional expertise and insights from such representatives but follow-on activities with PPI representation are planned.

## Conclusions

The PISA taxonomy can be successfully applied to patient safety incident reports to support the first stages in deriving learning and identifying areas for further enquiry. We identified no incidents that warranted new codes to be added to the PISA classification system, which may extend to other substantive public health crises, negating the need for additional, specific coding within such classification systems and related frameworks for similar system-wide constraints.

### Electronic supplementary material

Below is the link to the electronic supplementary material.

Supplementary Material 1

## Data Availability

The datasets used and/or analysed during the current study are available from previously published studies (COPE/ PSI RECORd). All data generated or analysed during this study are available from the authors upon reasonable request.

## List of abbreviations

WHO: World Health Organization
ICPS: International Classification for Patient Safety
GP: General Practitioner
PISA: PatIent SAfety (PISA) research group (Cardiff University)
