# Supplementary material for: Analysis of applying a patient safety taxonomy to patient and clinician-reported incident reports during the COVID-19 pandemic: a mixed methods study
Source: BMC Med Res Methodol. 2023 Oct 14;23:234. doi: 10.1186/s12874-023-02057-6 (PMC10576389; doi:10.1186/s12874-023-02057-6)
Supplement: Supplementary file 1 — Supplementary Material 1 [file 12874_2023_2057_MOESM1_ESM.docx]

Appendix 1. Expert Group Schedule

| Welcome & introduction | All |
| --- | --- |
| Presentation of the study, aims and objectives, and purpose of expert group | AC & TP |
| Group discussion:   - Does this difference in reported incidents and quality of reporting between patients and healthcare professionals reflect the experience of the participants? - How do you encourage patients to report safety incidents? | All |
| Example 1 presented | AC & TP |
| Group discussion:   - Are further COVID-related codes needed?   - More specific infection control codes? - Can we think of different contexts where these codes may also apply? | All |
| Example 2 presented | AC & TP |
| Group discussion:   - Are further COVID-related codes needed?   - Outcome COVID-19 or healthcare acquired infection? - Can we think of different contexts where these codes may also apply? | All |
| Example 3 presented | AC & TP |
| Group discussion:   - Are further COVID-related codes needed?   - Contributory factor patient behaviour – fear of COVID delaying presentations/not following advice - Can we think of different contexts where these codes may also apply? | All |
| Example 4 presented | AC & TP |
| Group discussion:   - Are further COVID-related codes needed?   - Contributory factor working conditions – specific remote consulting codes needed? - Can we think of different contexts where these codes may also apply? | All |
| Example 5 presented | AC & TP |
| Group discussion:   - Are further COVID-related codes needed?   - Contributory factor working conditions – remote consulting - Can we think of different contexts where these codes may also apply? | All |
| Example 6 presented | AC & TP |
| Group discussion:   - Are further COVID-related codes needed?   - Contributory factor service unavailable/policies – due to COVID disruption - Can we think of different contexts where these codes may also apply? | All |
| Example 7 presented | AC & TP |
| Group discussion:   - Are further COVID-related codes needed?   - Staff outcome – additional GP workload - Can we think of different contexts where these codes may also apply? | All |
| Summary and close | AC & TP |
